# Comparison of In Vivo and In Vitro Digestibility in Rabbits

**DOI:** 10.3390/ani11113267

**Published:** 2021-11-15

**Authors:** Sonia Tassone, Riccardo Fortina, Sabah Mabrouki, Yasser Hachana, Salvatore Barbera

**Affiliations:** 1 Department of Agriculture, Forestry, and Food Sciences, University of Turin, 10095 Grugliasco, Italy; sonia.tassone@unito.it (S.T.); mabrouki.sabeh@gmail.com (S.M.); salvatore.barbera@unito.it (S.B.); 2 Department of Animal Production, Higher Agronomic Institute of Chott-Mariam, University of Sousse, Susa 4042, Tunisia; hachana@yahoo.fr

**Keywords:** rabbit in vitro digestibility, Ankom Daisy^II^ Incubator, enzymatic technique, fecal inoculum

## Abstract

**Simple Summary:**

We tested the Ankom Daisy^II^ Incubator as an alternative to the in vivo procedures for measuring the in vitro dry matter digestibility of diets for rabbits. The results were variable according to the methods. Among those tested in the trial, the enzymatic one was more effective than those based on fecal inocula. We conclude that the Ankom Daisy^II^ Incubator can be used to estimate the in vivo dry matter digestibility of rabbit diets, but the choice of the method proved to be fundamental for the reproducibility of results.

**Abstract:**

The apparent dry matter digestibility of diets for rabbits was measured in vivo (ADMD_vv_) and in vitro with the Ankom Daisy^II^ Incubator. Four diets were tested: low fiber (LF), LF + 5% of pregerminated fenugreek seeds (PGFS) (LF5), LF + 10% PGFS (LF10), and high fiber (HF). For the ADMD_vv_, feces samples were collected from 56 White New-Zealand × Californian rabbits fed the 4 diets; animals were randomly allocated into 4 groups and housed in individual cages. For the in vitro trial, 3 methods were tested: fecal inoculum (FA) with Kansans State buffer; fecal inoculum with artificial saliva (FB); and multienzyme (ENZ). Fecal inocula were collected at slaughtering from the distal colon of rabbits fed ad libitum the LF diet. For FA and FB methods, the digestibility was measured at 36 and 48 h. The in vitro methods ranked the apparent dry matter digestibility of diets in the same order as in vivo, but ENZ values were always higher than FA and FB at 36 and 48 h. The prediction equations of in vivo digestibility with the ENZ method showed higher coefficient of determination (R^2^ = 0.69) and lower SE (0.190) than FA and FB; also, reproducibility was higher with ENZ (CV = 3.1%). In conclusion, different methods can be applied to the Ankom Daisy^II^ Incubator to study the digestibility in rabbits. In our trial, the better reproducibility was observed with the multienzyme method than FA and FB were probably related to the variability of inocula.

## 1. Introduction

The evaluation of feed digestibility is an important issue on which much research is focusing using different methods and techniques. In vitro techniques have gained considerable importance since the increased interest in animal well-being [1]. In vitro digestibility studies on rabbits are based on multienzymes methods or methods that use cecal or fecal inocula. Ramos et al. [2] verified the good accuracy of the multienzyme method used for pigs [3] to predict dry matter digestibility in rabbits. This method was based on three incubation steps with pepsin, pancreatin and microbial fiber-degrading enzymes. Fernández-Carmona et al. [4] described a new in vitro method for rabbits based on the procedure developed by Lindgren [5] for ruminants, and by Löwgren et al. [6] for pigs, using cecal content and artificial saliva. The results showed a high correlation (R^2^ = 0.99) with in vivo digestibility data. Pascual et al. [7] compared the techniques of other authors [4,8] and found that the multienzyme method showed significantly better results. Since then, this method has been widely used for digestibility trials in rabbits [8,9,10]. Carabaño et al. [9] found adequate repeatability and reproducibility within and between laboratories of the three-step incubation method of Ramos et al. [2]. They modified and applied the multienzyme method to the Ankom Daisy^II^ Incubator, a versatile instrument effective in reducing working times that can be used with different types of inocula or enzymes [11]. This instrument has been used by De Blas et al. [12] to determine the digestibility of co-products derived from olive cake in rabbit diets. Ferreira et al. [13,14] modified the procedure described by Abad et al. [15] and measured the digestibility with the Ankom Daisy^II^ Incubator using the cecal content for the last multienzyme step. The Daisy^II^ Incubator was also tested by Kovitvadhi et al. [16] for the estimation of the in vitro apparent dry matter digestibility, with positive results using crude enzymes extracted from the digestive organs of rabbits.

Even if the Ankom Daisy^II^ Incubator and the multienzyme methods are commonly used for in vitro digestibility trials in rabbits, other methods could be adapted to this instrument. The use for example of feces as inoculum for in vitro digestibility could be an alternative for rabbits. Different authors have already demonstrated on horses [17,18,19,20,21] and donkeys [22,23]—the closest livestock species to rabbits—that feces are a suitable source of microbial inoculum for in vitro digestibility studies. In particular, Earing et al. [20] demonstrated that when equine feces are used as inoculum for the in vitro methodology developed for the Ankom Daisy incubator, it could produce accurate estimates of in vivo equine dry matter and NDF digestibility, particularly with an incubation period of 72 h.

The objective of this study was to test different methods to determine the in vitro apparent dry matter digestibility of diets for rabbits with the Ankom Daisy^II^ Incubator, and to compare the results with the in vivo digestibility values.

## 2. Materials and Methods

### 2.1. Diets

The diets were formulated for growing rabbits with low or high fiber content and fenugreek seeds. Fenugreek (*Trigonella foenum-graecum*) is an annual legume widely cultivated in Tunisia as a spice and forage crop. The seeds contain approximately 31% and 21% of soluble and insoluble dietary fiber, respectively; the main component of the soluble fraction is a galactomannan that can be extracted with water and ethanol (fenugreek seed gum). This galactomannan is highly fermentable and is a potential prebiotic due to his high fermentability and resistance to acid and pancreatic digestion [24]. For the in vivo and in vitro trials, a control diet with low fiber content (LF) and 3 experimental diets with or without pregerminated fenugreek seeds (PGFS) were used: LF + 5% PGFS (LF5); LF + 10% PGFS (LF10); high fiber (HF). LF diets had similar crude protein (CP) and Neutral Detergent Fiber (NDF) amount; HF diet had higher NDF, Acid Detergent Fiber (ADF), cellulose and lignin (ADL), and lower CP and non-fiber carbohydrates (NFC) than LF diets. Composition and characteristics of the 4 diets are reported in Table 1. For chemical analyses, samples were grounded using a cutting mill (MLI 204; Bühler AG, Uzwil, Switzerland). Dry matter (DM), ash, CP) and ether extract (EE) were determined according to AOAC [25], as described by Fortina et al. [26]. Fiber fractions (NDF, ADF and ADL) were determined using the Ankom^200^ Fiber Analyzer (Ankom Technology, Macedon, NY, USA) following the procedures of Mertens [27] and Van Soest et al. [28]. For in vitro digestibility, ground samples were weighed (0.50 ± 0.05 g) into F57 bags; two empty bags were used as blanks.

### 2.2. In Vivo Digestibility (ADMD_VV_)

The trial was conducted on fifty-six White New-Zealand × Californian rabbits (age: 32–35 days; live weight (LW): 530–580 g) and replicated two times. Animals were randomly allocated into 4 groups and housed in individual cages (30 × 30 cm) made of galvanized wire (mesh size: 1.5 cm), equipped with automatic drinkers and feeders. During the trial the temperature ranged from 18 to 24 °C. The 4 diets were offered *ad libitum* for 10 days of adaptation period. During the collection period (4 days) feed intake was recorded individually and calculated as the difference between the initial weight of the feed and back orts. Feces were collected every day, in the morning, weighed and immediately frozen. Dry matter of feces was assessed following a two-step procedure; in the first, approximately 50% of each sample was dried at 50 °C for 36 h; in the second step, 100 g of pre-dried samples were oven dried at 103 °C for 24 h. Data were pooled and the in vivo apparent DM digestibility (ADMD_VV_) for each diet was calculated with the formula reported in Section 2.4.

### 2.3. In Vitro Digestibility (ADMD_AD_^II^)

Three methods were used to estimate the in vitro apparent DM digestibility with the Ankom Daisy^II^ Incubator (Ankom Technology Corporation Fairport, New York, NY, USA). The analyses were conducted according to Tassone et al. [11] using faecal inocula (FA and FB methods) and multienzymes (ENZ method). Faecal inocula were collected in a slaughterhouse of Rivoli (Italy) from approximately 120 White New-Zealand × Californian rabbits fed *ad libitum* the control (LF) diet. Immediately after slaughtering, the feces were taken manually from the distal colon, placed in thermal bottles oxygen free, kept at 39 °C and immediately transferred to the lab (15–20 min travel). 

The first method (FA) is a modification of the technique described by Earing et al. [20] for horses. The fecal inoculum was prepared mixing 200 g of rabbit feces and 400 mL of warmed buffer solution for 30 s under CO_2_ (Osterizer Cyclo-Trol Eight; Oster, Moncalieri, Italy). The buffer solution was prepared mixing 1500 mL of A solution (10 g/L of KH_2_PO_4_, 0.5 g of MgSO_4_ × 7H_2_O, 0.5 g/L of NaCl, 0.1 g/L of CaCl_2_ × 2H_2_O and 0.5 g/L of CH_4_N_2_O) and 300 mL of B solution (15 g/L of Na_2_CO_3_ and 1 g/L Na_2_Sx9H_2_O). The inoculum (400 mL) was filtered through a double-layered cheesecloth into each jar containing F57 filter bags and 1800 mL of pre-warmed buffer solution (40 °C). After incubation (36 and 48 h), the solution was discarded, and bags rinsed with cold water. Samples were dried at 103 °C for 24 h and weighed.

The second method (FB) is a modification of the technique described by Pascual et al. [7]. The fecal inoculum was prepared mixing 200 g of rabbit feces and 320 mL of artificial saliva (0.5 g MgSO_4_ + 7H_2_O, 1.5 g/L NaCl, 3.8 g/L NaHCO, 4 g/L K_2_HPO_4_ and 0.5 g (NH_4_)_2_HPO_4_) under CO_2_; after 1 h, the solution was centrifuged for 5 min at 3500 rpm. The supernatant was added into a jar containing F57 filter bags and 1680 mL of pre-warmed artificial saliva solution. After incubation (36 and 48 h), samples were treated as previously described. 

The third method (ENZ) is a modification of the technique described by Abad et al. [15]. Digestion of samples occurred in 3 steps as following:

- Step 1 (Pepsin digestion): 600 mL of phosphate buffer solution (0.1 M, pH 6) were added to a jar containing 240 mL of HCl (0.2 M); the pH was adjusted to 2 using HCl or NaOH (1 M). After 30 min at 40 °C, 0.6 g of pepsin from porcine gastric mucosa (Sigma-Aldrich, Oakville, Ontario, CA, Canada) were added. Samples were incubated for 90 min at 39 °C.
- Step 2 (Pancreatin digestion): 240 mL of phosphate buffer solution (0.2 M, pH 6.8) were added to a jar containing 120 mL of NaOH (0.6 M); the pH was adjusted to 6.8 with HCl or NaOH (1 M). Immediately after, 2.4 g of pancreatine (P-1750, Sigma-Aldrich) were added to the jar. Samples were incubated for 210 min at 39 °C.
- Step 3 (Viscozyme digestion). After step 2, the pH was adjusted to 4.8 with 30% acetic acid solution. Viscozyme from *Aspergillus aculeatus* (Novozymes, Bagvaerd, Denmark) (12 mL) was added and samples incubated at 40 °C. After 16 h, the solution was discarded and the bags were rinsed with distilled water, ethanol 95% (5 min) and acetone (5 min). Bags were oven-dried at 103 °C for 24 h, then weighted.

Each method was replicated for three times, and each diet sample was weighed in triplicate. In vitro digestibility values were calculated as described in the next paragraph (Section 2.4) and compared with the results of an in vivo digestibility trial.

### 2.4. Calculations

The in vivo DM digestibility (ADMD_VV_) was calculated as follows:

ADMD_VV_ (% DM) = 100 × (DM_i_ − DM_e_/DM_i_)

where:

DMi (%) = ingested DM
DMe (% DM) = excreted DM.

The in vitro apparent DM digestibility with the Ankom Daisy^II^ Incubator was calculated as follows:

ADMD_FA, FB, ENZ_ (% DM) = 100 − (W_3_ − (W_1_ C_1_)) × 100/W_2_ × DM

where:

C_1_ (%) = blank bag correction (final oven-dried weight/original blank bag weight)
W_1_ (%) = bag tare weight
W_2_ (%) = sample weight
W_3_ (%) = final bag weight after in vitro incubation.

### 2.5. Statistical Analysis

Data were statistically analyzed by variance analysis using PROC GLM of SAS (Statistical Analysis System Institute, Cary, NC, USA, 2021) [29] adjusted by Tukey method. A unifactorial design was used and methods were analyzed separately for each diet. For each method, a linear regression analysis (PROC REG, procedure in SAS) was used to assess: (1) the relationships between in vivo and in vitro digestibility; (2) the regression equations for predicting the in vivo digestibility from each in vitro method. Reproducibility of the methods was calculated as reported by Tassone et al. [23] and it was expressed as coefficient of variation (SD/mean × 100).

## 3. Results

Table 2 shows the effects of methods (FA and FB at 36 and 48 h incubation time, and ENZ) on in vitro digestibility of the 4 diets. Regardless the method used, the HF diet showed low digestibility values due to the high amount of NDF, ADF, and lignin. ADMD_ENZ_ was always similar to in vivo values. The ENZ method yielded higher degradability of samples than FA and FB at 36 or 48 h. Among fecal methods, time had no effect on digestibility. The digestibility of LF5, LF10 and HF diets were similar at 36 or 48 h of incubation; only for the LF diet, the digestibility with FA_36_ was statistically different from FB_48_.

**Figure 1:**
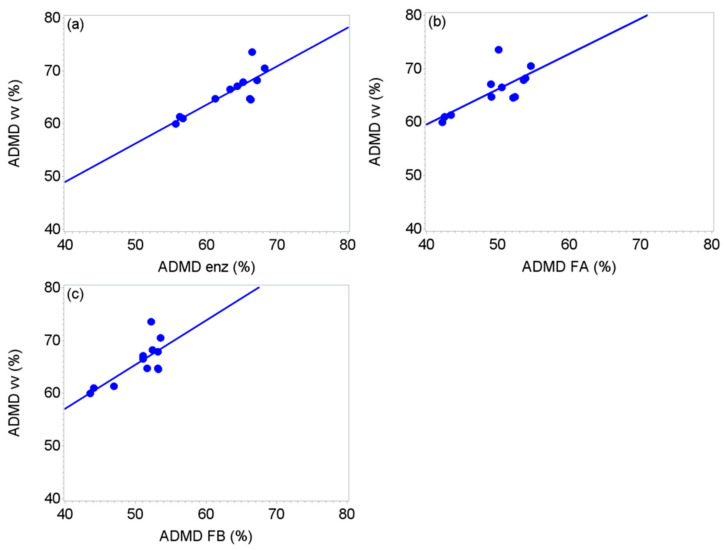
Relationship between in vivo digestibility (ADMD_VV_) and 36 h in vitro digestibility using the Daisy^II^ Incubator (ADMD) with different methods: ENZ (**a**), FA (**b**) and FB (**c**). Abbreviations: ADMD VV, apparent in vivo dry matter digestibility; ADMD enz, apparent in vitro dry matter digestibility using enzyme method; ADMD FA, apparent in vitro dry matter digestibility at 36 h using FA method; ADMD FB, apparent in vitro dry matter digestibility at 36 h using FB method.

The prediction equations obtained for estimation of in vivo digestibility from in vitro techniques were all statistically significant (Table 3 and Figure 1). The equations obtained with the ENZ method predicted the in vivo digestibility (ADMD_VV_) with higher precision (R^2^ = 0.69) and lower SE (2.313) than FA and FB. Reproducibility was higher with multienzyme than fecal methods, that in general gave poorer results for reproducibility than ENZ. Using the technique described by Earing et al. [20], the method (FA) showed better SE values than FB at 36 and 48 h of incubation; the highest precision was observed at 36 h for FA_36_ and FB_36_ (R^2^ = 0.55 and 0.57, respectively). At 36 h FA also showed the best reproducibility (Rep = 3.9) among fecal methods. 

## 4. Discussion

### 4.1. Fenugreek Seeds Inclusion

In our trial, two diets were added with small amounts (5 and 10%) of fenugreek seeds, rich in soluble dietary fiber. Rodríguez-Romero et al., [30] demonstrated that NDF reduces fecal digestibility, whereas neutral detergent soluble fiber promotes better conditions for cecal fermentation. Some authors [31,32,33] proposed the increasing of the dietary soluble fiber content as a strategy to improve the integrity of the intestinal mucosa and modulate the intestinal microbiota of rabbits. Considering that easily fermentable fiber is partially fermented in the small intestine [34,35], different authors [31,36,37,38] argued that just when it is included in moderate proportions it can stimulate cecal fermentation and microbial nitrogen, recycling through an increase in cecal weight. An alternative to a high inclusion of soluble fiber-rich raw materials, which also increases other fibrous fractions or could increase the amount of protein linked to fiber, is the direct inclusion of a small amount of an isolated soluble fiber, acting as a prebiotic. Some works have been done [39,40,41,42,43], but no consistent results were found. In our trial, with the inclusion of 5% of fenugreek in the diet, no effects were observed on apparent digestibility, but the inclusion of 10% showed the tendency of increasing the digestibility. Zemzmi et al. [44] found that the dietary inclusion up to 0.5% of fenugreek seeds gum (a potential prebiotic extracted from seeds, containing a galactomannan resistant to acid and pancreatic digestion and highly fermentable) did not significantly affect the apparent digestibility of main nutrients and main cecal environment traits, but it lowered the cecal pH and it can be used by the fibrolytic microbiota of the rabbit’s caecum. Martinez-Vallespín et al. [45,46] demonstrated that high fermentability of galactomannans in the cecum could promote the fibrolytic microbiota, influencing the use of the rest of the fibrous fractions. Majeed et al. [24] showed that water-soluble fiber, like the galactomannan of fenugreek seed gum, are highly fermentable in the rabbit caecum and can lead to high gas production and VFAs. Moreover, the higher microbial activity associated with the increased availability of a fermentable substrate is also usually associated with promoted microbial protein synthesis and reduced N-NH_3_ level in the cecum [33].

### 4.2. Digestibility Trial

The digestibility values observed in the trial were in the range reported for rabbit diets [9]. Higher values were observed in vivo than in vitro, as already found in rabbit [7], in horse [20] and donkey [23] respectively. Statistical differences were observed with the fecal inoculum only, while with the multienzyme the results were similar to the in vivo data. Pascual et al. [7] observed similar results for single feeds, comparing the DM digestibility using multienzyme, or cecal and fecal inocula. They reported higher precision with multienzyme and cecal method (R^2^ = 0.95 and 0.88, respectively), especially for fibrous feeds. In the present study we used feces as an alternative source of inoculum to the cecal fluid, as reported by other authors Bovera et al. [47], who evaluated the changes in cecal fermentations of feeds or diets for rabbits using feces as inoculum [48]. The authors found significant equations of regression for estimating some important cecal fermentation parameters using feces as inoculum and concluded that rabbit feces are a valid alternative to cecal inoculum for gas tests or to estimate the cecal fermentation of roughages. However, this result is not confirmed by Pascual et al. [7], who demonstrated that the cecal inoculum was better than the fecal, albeit a greater standardization was necessary. In our trial the use of the Ankom Daisy^II^ Incubator allowed to standardize the analytical procedures; samples were incubated together, and the filter bag-based technique is generally assumed to have conditions within bags similar to the conditions in the surrounding environment. In our study, the lowest CV value (3.9%) was observed for the FA method at 36 h of incubation. The use of an enzymatic method in this trial was also dictated by the need to avoid being dependent on animals. We measured the precision of a method (ENZ) that used only commercial enzymes, avoiding the use of cecal content in the last step of the digestion trial, as described by Ferreira et al. [14]. According to Pascual et al. [7], using only enzymes for in vitro digestion of single feeds, the results showed a higher correlation with in vivo values (R^2^ = 0.95). Similar results were obtained in our study, but with a lower correlation (R^2^ = 0.69). This result could be explained with higher variability of our results (SE > 2) than reference method (SE 0.7) [7]. Pascual et al. [7], demonstrating that the prediction equations for DM digestibility obtained with the multienzyme technique had the highest precision and lowest variability when compared to the method based on cecal and fecal inocula. In addition, Carabaño et al. [9] reported adequate repeatability and reproducibility of the three-step incubation multienzyme method compared to in vivo method. Fernández-Carmona et al. [4] observed a high correlation (R^2^ = 0.99) between in vivo and in vitro digestibility in rabbits using cecal content and artificial saliva

The rabbit digestive system is characterized by the importance of the role of the caecum [30]; the fibrous fraction of the diet is the main determinant of cecal fermentation [14]. The most important factor regulating the rate of passage and microbial activity in the digestive system of rabbits is the slowly fermentable dietary fiber [36,49]. It decreases mean retention time, favoring cecal turnover; its low fermentability contribute to impairing the rate of microbial protein synthesis [36]. The source of fiber helps to determine the extent [50,51] and diversity [31] of cecal microbial proliferation, thus contributing to the prevention of digestive pathologies [52]. However, this response largely also depends on the fiber type, and its degree of lignification [53]. In our trial, the diet with high fiber content (HF) and high lignin, always showed lower digestibility values than LF diets, both in vivo and in vitro. For the fecal methods, we used two different incubation times (36 and 48 h), but results did not show any statistical difference. According to Pascual et [7], a 36 h of incubation time proved to be sufficient for the estimation of the digestibility. Regardless the method used, in this trial we confirmed the negative correlation between ADMD and NDF, ADF, lignin and cellulose, in accordance with other authors [10,12].

## 5. Conclusions

Introduced for rumen fermentation studies, the Ankom Daisy^II^ Incubator has been adapted with success also to monogastric. This trial confirms that it is a reliable instrument for repeatable and relatively low-cost digestibility trials, also applicable to evaluate the in vitro apparent digestibility of diets for rabbits using different methods.

The results of the trial showed that in vivo values were higher than in vitro estimates. However, the 3 in vitro methods ranked the ADMD of diets in the same order as in vivo. The multienzyme method showed a better repeatability than the fecal methods, but the prediction equations showed low precision for both. Based on these results, it would be convenient to carry out more in vitro digestibility studies reducing the variability of fecal inocula; studies should be also aimed to better understand the fermentation pattern of soluble fibers included in rabbit diets and their role on rabbit performances.

## Figures and Tables

**Table 1:** Ingredients and chemical composition of diets.

	LF	LF5	LF10	HF
**Ingredients (%)**				
Pre-germinated fenugreek seed	0	5	10	0
Alfalfa meal	10	5	0	32
Barley	25	25	25	9
Soybean meal	10	10	10	9
Wheat bran	30	40	40	30
Soybean husk	10	10	10	13
Beet molasses	3	3	3	3
Animal fat	0	0	0	2
Minerals and vitamins	2	2	2	2
**Chemical composition (% DM)**				
Dry matter (DM)	87.8	87.5	87.4	88.3
Organic matter (OM)	81.3	81.4	81.7	80.6
Crude protein (CP)	17.8	18.3	18.5	17
Ether extract (EE)	3.4	4	4.4	5.2
Neutral detergent fiber (NDF)	35.6	35.2	34.8	45.1
Acid detergent fiber (ADF)	15.8	15.3	14.2	24.5
Lignin (ADL)	3.4	3.3	3.1	5.4
Hemicellulose	19.8	19.9	20.6	20.6
Cellulose	12.4	12	11.1	19.1
Non fiber carbohydrates (NFC)	35.9	35.5	35.8	24.3

LF: low fiber diet; HF: High fiber diet.

**Table 2:** Effect of method on the dry matter digestibility of diets.

Diet	ENZ	FA_36_	FA_48_	FB_36_	FB_48_	VV	SEM
LF	64.2 ^a^	51.5 ^c^	53.4 ^bc^	52.6 ^bc^	55.2 ^b^	65.7 ^a^	2.34
LF5	64.6 ^a^	50.6 ^b^	53.6 ^b^	51.8 ^b^	54.5 ^b^	66.1 ^a^	3.19
LF10	67.3 ^a^	52.9 ^b^	54.0 ^b^	52.7 ^b^	56.1 ^b^	70.7 ^a^	2.63
HF	56.4 ^a^	42.7 ^c^	43.4 ^c^	44.8 ^c^	45.1 ^c^	60.8 ^a^	2.56

^a–c^, =*p* < 0.01 on the same row within each diet. Abbreviations: LF, low-fiber diet; LF5, low fiber diet with 5% of fenugreek seed; LF10, low fiber diet with 10% of fenugreek seed; HF, high-fiber diet. FA_36_, fecal method A at 36 h; FA_48_, fecal method A at 48 h; FB_36_, fecal method B at 36 h; FA_48_, fecal method B at 48 h; ENZ, multi-enzyme method; VV, in vivo method.

**Table 3:** Regression equations and reproducibility (CV,%) of the different in vitro digestibility methods (*n* = 24).

Method	Time (h)	Y	A	B	X	R^2^	SE	*p*	Rep
FA	36	ADMD_VV_	33.063	0.662	ADMD_FA36_	0.55	2.804	0.006	3.9
	48	ADMD_VV_	39.045	0.5242	ADMD_FA48_	0.49	2.988	0.012	5.4
FB	36	ADMD_VV_	23.641	0.8353	ADMD_FB36_	0.57	2.745	0.005	5.3
	48	ADMD_VV_	36.022	0.5649	ADMD_FB48_	0.48	3.001	0.012	5
ENZ		ADMD_VV_	19.802	0.729	ADMD_ENZ_	0.69	2.313	0.001	3.1

Abbreviations: ADMD_VV_, in vivo apparent dry matter digestibility; ADMD_FA36_, apparent dry matter digestibility with FA method at 36 h of incubation; ADMD_FA48_, apparent dry matter digestibility with FA method at 48 h of incubation; ADMD_FB36_, apparent dry matter digestibility with FB method at 36 h of incubation; ADMD_FB48_, apparent dry matter digestibility with FB method at 48 h of incubation; ADMD_ENZ_, apparent dry matter digestibility with ENZ method; R^2^, coefficient of determination; Rep, reproducibility expressed by coefficient of variability.

## Data Availability

Data are available in a publicly accessible repository.
